# Surgical Aid in Chronic Ulcer of the Stomach

**Published:** 1908-09

**Authors:** J. A. Nixon

**Affiliations:** Physician to the Bristol Royal Infirmary


### SURGICAL AID IN CHRONIC ULCER OF THE STOMACH.

BY

J. A. Nixon, M.B. Cantab., M.R.C.P. Lond.

*Physician to the Bristol Royal Infirmary.*

A constant succession of dyspeptics, some alcoholic, some phthisical, all full of strange complainings in terms borrowed for the most part from soul-stirring newspaper advertisements, passing rapidly through an out-patient clinic is calculated to blunt one's interest, and to lull one into the passive belief that none are ever cured, and that the majority of them may be relieved by copious irrigation of the stomach with concoctions of rhubarb and gentian. Invaluable as this remedy proves in a great number of cases, it cannot be regarded as a panacea for every variety of digestive disturbance, and of late years its pre-eminence has been challenged by the surgeon. The operative treatment of chronic gastric ulcer has been attended by such noteworthy results, that the physician is tempted sometimes not to discriminate too closely, but to submit any and every case of indigestion to operation, with the inevitable penalty of disappointment.

Whether gastro-enterostomy in one of its forms is the best operation that can be devised, or whether some better procedure will presently be introduced, is not the object of discussion in this paper. So far as it goes it is a good operation, it relieves the patient under certain conditions; and the duty of the physician (in the intervals of suggesting fresh plans for operation to the surgeon) is to endeavour by every means in his power to select those cases that are likely to benefit from surgical interference, and learn to know those in which no good results are likely to follow.

The cases under my care which have been operated upon, in the belief that a chronic ulcer of the stomach or duodenum was present, are shown in the following list.

Ten cases suspected of chronic ulcer do not form a large series from which to arrive at general conclusions, but they provide illustrations of the difficulties encountered in diagnosing and prognosticating the conditions which surgery will relieve.

**Case 1.**—T.C.G., a man, aged 53. Eleven years previously suffered from vomiting (daily for three weeks), with a single large hæmatemesis. Six years ago treated for gastric ulcer, and again one year ago when he vomited frequently, sometimes "coffee grounds." No pain or tenderness, no gastrectasis, some indefinite resistance in epigastrium. Argyll-Robertson pupils, knee-jerks present, no inco-ordination, analgesia in arms and legs. Free HCl absent, and lactic acid (0.129 per cent.) present in gastric juice.

Here there seemed so much room for doubt, that in spite of recognising the fact that the man was probably suffering from spinal sclerosis, operation was advised. It was possible that there was some old ulcer or carcinoma of the stomach, and the general condition of the patient was fair. An exploration was made, no ulcer or growth was found, gastro-enterostomy was performed. Three months later he was well except for slight fulness after meals. Subsequently the vomiting recurred temporarily. The conclusion is that these attacks are gastric crises, yet there was good reason for believing it might be otherwise, and the patient suffered no ill-effects from his operation.

**Case 2.**—A.H., a man, aged 32. Four years ago was warded for a month on account of vomiting blood and diarrhœa, one year ago a similar attack lasting six weeks. A fortnight before admission he had vomiting with blood. Vomiting always occurred soon after food. Painful spot in the nipple line just below left hypochondrium. Extreme emaciation. No signs of other disease.

At the operation no ulcer, growth or adhesions were found, but gastro-enterostomy was performed. Immediately the man improved and put on flesh (this had not been the case under medical treatment), but he has relapsed several times. Once he developed an ulcer of the larynx, and several times since he has had numerous small superficial ulcers in the mouth, suspected variously of being syphilitic or tuberculous. On the last occasion he came under observation it was evident that these ulcers started as blebs. Perhaps pemphigus is their true origin. Still, the improvement after operation was remarkable, and the patient has never seen reason to regret its performance.

**Case 3.**—M.C., a girl, aged 14, who had persistent vomiting after food with so much epigastric tenderness and pain of paroxysmal character that, although she had a few pleural crepitations at her right base, it was decided to perform an exploratory laparotomy. She urged us to operate, the pain was so severe, and she was so sure of its localisation.

Mr. Carwardine explored, and found an abnormal attachment (? congenital) of the omentum to the anterior abdominal wall; this he divided, but did not open the stomach. There was no external sign of ulcer. Eventually she developed extensive diffuse phthisis, and her pain was in no way relieved. Doubtless this pain was connected with a diaphragmatic pleurisy.

**Case 4.**—F.C., a girl, aged 19, with a history of six years' indigestion, constant pain relieved by lying down; vomiting at no definite time after meals; three years ago a single hæmatemesis; slight epigastric tenderness, no gastrectasis. She described her pain as so severe, so localised, and as making her life such a burden, that she begged for an operation "to see if there was not something there." Medical treatment had always failed to do any good.

Mr. Munro Smith operated, and found a stomach firmly adherent on its posterior aspect to a mass feeling the size of a cricket ball, which presented no prospect of safe removal. Gastroenterostomy was performed. There was complete relief from pain, no recurrence; the girl put on flesh; two and a half years afterwards could eat "just anything," and was happily married.

**Case 5.**—L.D., a girl, aged 18, with the appearance of a fairly healthy child of 12 (Lorain's infantilism). Epigastric pain, worse after food, for two years. Hæmatemesis (Oj) nine months ago, and one month later. Great cutaneous hyperæsthesia in epigastrium. Enormous gastrectasis, greater curvature just over pubes. Had menstruated once at 13, absent since.

Gastro-enterostomy performed by Mr. Carwardine. Inflammatory tumour found, completely obstructing pylorus. The relief was immediate, the child grew into a woman in a few weeks. The stomach remained distensible for many months, and at rare intervals there was vomiting. The weight increased in eight months from 6 st. to 10 st. 3 lb. After six months the stomach could no longer be abnormally distended with seidlitz powder. Eighteen months after operation was in excellent health and appeared a healthy young woman of 20; catamenia regular.

**Case 6.**—A.H., single woman, aged 34. Indigestion on and off since childhood. Hæmatemesis three years ago. Pain directly after food, temporarily relieved by medical treatment. No vomiting, no tenderness, no gastrectasis.

Laparotomy by Mr. Carwardine. Hour-glass stomach, also scarred duodenal ulcer obstructing pylorus. Gastro-jejunostomy and gastro-gastrostomy performed. The relief from operation was considerable, but at times indigestion returns (eighteen months after operation). The patient is very glad she was operated on.

**Case 7.**—M.V., married woman, aged 37. Fourteen years ago vomiting after food with streaks of blood; indigestion on and off ever since. Pain directly after food in epigastrium and back. Water-brash, gastrectasis, lump felt in epigastrium. Gastric juice, free HCl, no lactic or butyric acid.

Gastro-enterostomy by Mr. Carwardine. Duodenal adhesions and considerable dilatation of stomach found (? duodenal ulcer). Marked relief of indigestion and water-brash, continuing for two years.

**Case 8.**—C.G., single woman, aged 37. Frequently under treatment for dyspepsia during past sixteen years. Pain about one hour after meals, relieved by vomiting. Hæmatemesis eight years and six years ago. In latter years vomiting less frequent, but the desire to vomit has increased. Superficial cutaneous hyperæsthesia and some deep tenderness in epigastrium. No gastrectasis.

Gastro-enterostomy performed by Dr. J. Swain. Pylorus not apparently thickened, no ulcer found, but very great relief from the operation. The patient is a maid at the Bristol Royal Infirmary, and is under constant observation. Her satisfaction ten months after operation is undiminished.

**Case 9.**—K.S., married woman, aged 46. For twelve months had complained of pain just below left hypochondrium one hour after food. Sickness after every meal, which used to relieve the pain, but later ceased to do so. Always free from pain when constipated or when lying down. Never hæmatemesis. Slight tenderness in epigastrium, no gastrectasis.

Operation by Mr. Bush. Marked puckering along lesser curvature of stomach, an indurated mass involving equally anterior and posterior surfaces, and reducing the length of lesser curvature to one half of normal. Gastro-enterostomy performed with complete and lasting relief (one year later).

**Case 10.**—J.M., man, aged 55. For five years pain after food, vomiting (a pailful at a time) for twelve months. Progressive wasting, lemon tint of skin noticed by friends. Gastrectasis, visible peristalsis, lesser curvature just below umbilicus. A firm "knob" felt at right edge of right rectus when peristalsis reached this point. Liver dulness displaced downwards but not enlarged. Gastric juice: Free HCl absent.

Operation by Mr. Hey Groves. Pylorus thick and nodular, anterior surface marked by scar to which omentum adhered: complete pyloric stenosis. Gastro-enterostomy performed and piece of tumour removed for examination. *Pathol. report.*—At first only inflammatory tissue found, but later in posterior pyloric wall undoubted area of columnar-celled carcinoma. Twelve days after first operation Mr. Groves performed pylorectomy. The patient made a good recovery and experienced great relief from the operation (after six months).

### ANALYSIS OF CASES.

| Case. | History. | Pain. | Vomiting. | Hæmatemesis. | Dilatation. | Condition found. | Result. |
|---|---|---|---|---|---|---|---|
| 1.—T.C.G. .. | 11 years .. | No .. .. | Yes. Frequent .. | Yes. Twice .. 11 and 6 years ago | No .. | No ulcer found .. | Temporary Relief. |
| 2.—A.H. .. | 4 years .. | Constant .. | Yes. After food . | Yes. Twice .. | No .. | No ulcer found .. | Partial relief. |
| 3.—M.C. .. | 6 months .. | Paroxysms | Yes .. .. .. | No .. .. .. | No .. | Phthisis .. .. .. | No relief. |
| 4.—F.C. .. | 6 years .. | Constant .. | Yes. Without relation to food | Yes. Once .. .. | No .. | Old gastric ulcer with adhesions | Relief. |
| 5.—L.D. .. | 2 years .. | After food . | Rarely .. .. .. | Yes. Twice.. .. | Yes .. | Old pyloric ulcer with complete stenosis | Relief. |
| 6.—Annie H | From childhood | After food . | No .. .. .. | Yes. Once .. .. | No .. | Two ulcers. Gastric and duodenal | Relief. |
| 7.—M.V. .. | 14 years .. | After food . | Yes .. .. .. | No .. .. .. | Yes .. | Old duodenal ulcer with adhesions | Relief. |
| 8.—C.G. .. | 16 years .. | One hour after food | Yes .. .. .. | Yes. Twice .. | No .. | No ulcer or adhesions | Great relief. |
| 9.—K.S. .. | 1 to 2 years | One hour after food | Yes. After food . | No .. .. .. | No .. | Old ulcer on lesser curvature with much puckering | Relief. |
| 10.—J.M. .. | 5 years .. | After food . | Yes. A pailful at a time | No .. .. .. | Yes .. | Old pyloric ulcer ? carcinoma | Relief. |

Analysis of seven cases improved by operation :—

| | | |
|---|---|---|
| Pain present in | .. .. | 7 cases. |
| Vomiting present in .. | .. | 6 cases. |
| Hæmatemesis present in | .. | 4 cases. |
| Stenosis present in .. | .. | 3 cases. |

In discussing these cases one is tempted to look for some leading symptom or combination of symptoms, or to hope for some mechanical (chemical or otherwise) method of arriving at a diagnosis of chronic ulcer of the stomach. But excepting pain and vomiting there seems no other reliable sign, and these two are shared by many other diseases.

*Gastric contents.*—Little or no help is derived from the test-meal, and the presence or absence of this or that constituent. A considerable experience of "test-meals," carried out in various hospitals upon the subjects of many diseases, carries with it the conviction that "test-meals" offer absolutely no reliable division of gastric cases into those which will and those which will not benefit by operation. Hyperchlorhydria, hypochlorhydria, achylia gastrica, deficient or increased motility, will none of them give the unfailing indication.

*Gastrectasis* is by no means necessarily present in the cases which profit most by gastro-enterostomy.

*Tumour.*—The presence of a palpable tumour means very little in the diagnosis between inflammatory tissue and neoplasm ; its absence has no bearing on the question of a profitable or unprofitable operation.

*Hæmatemesis or Melæna.*—Both these signs are most misleading. Hæmatemesis in small quantities as described by the patient is most difficult to distinguish from hæmoptysis. Malignant disease, especially in the early stage, occasionally causes a single large hæmatemesis. Duodenal ulcer is not uncommonly associated with hæmatemesis and melæna from regurgitation of blood through the pylorus. The erosion of the liver by an adherent simple ulcer will give rise to constant "coffee-ground" vomiting.

Hæmatemesis from a general oozing of the gastric mucosa (gastrostaxis) is not unknown, may be very severe, and profits but little by gastro-enterostomy.

*Cutaneous hyperæsthesia,* especially galvano-hyperæsthesia, may be of great help in diagnosis. The absence of this sign need deter no one from operation if other indications are present.

The chief guide to operation is the patient as a whole; A long history of indigestion, with vomiting and occasional attacks of hæmatemesis dating from early adult life (I begin to think that gastric ulcer is not so very rare in children of 12 or 14) will form the foundation of most accounts of chronic ulcer. While to these may be added epigastric pain—pain before food, directly after food, an hour or two after food and pain all the time; the sufferers from ulcer of the stomach or duodenum know all these variations. A resistance, thickening or definite lump in the epigastrium only forms an additional reason for operating; and gastrectasis with visible peristalsis as ascertained by inflation with $CO_2$ clinches the matter.

Operation is indicated in:—

(*a*) Painful ulcer.
(*b*) Recurrent haemorrhage.
(*c*) Pyloric stenosis.

In these three conditions there will probably be no difference of opinion as to their suitability; but there is one more class, consisting of chronic dyspeptics, who, having honestly tried medical treatment, find themselves unrelieved. With the proviso that operation is not undertaken for the following conditions, they should be operated on:—

(1) Chronic alcoholism or drug-taking.
(2) Gastric crises.
(3) Phthisis.
    (*a*) Dyspepsia.
    (*b*) Hæmoptysis.
    (*c*) Diaphragmatic pleurisy.
(4) Mitral stenosis.
    (*a*) Pain.
    (*b*) Hæmoptysis.

(5) Cirrhosis of liver.
(6) Chronic Bright's disease (dyspepsia).
(7) Renal colic.
(8) Addison's disease (nausea, vomiting and faintness).
(9) Overloaded or distended colon.
(10) Arsenic poisoning.

*Atonic dilatation.*—Clifford Allbutt says that "the frequency of dilatation of the stomach as a complication or sequel of acute disease is not sufficiently appreciated" in the estimate of the causes of dilatation of the stomach not depending on pyloric stenosis, and he quotes (not in agreement) "a London physician of almost singular eminence," who said to him "that of dilatation of the stomach apart from pyloric obstruction he knew nothing." Yet except during convalescence from an acute disease, I believe the diagnosis of "atonic dilatation" should be received with the utmost scepticism. It has been quite astonishing to find how frequently the *post-mortem* or (in modern times) the operating table has shown an obstructive cause for the so-called atony. Unless such a case yields very quickly to diet and treatment it should be explored.

For general neurotic symptoms coexisting with a dilated stomach no great respect is demanded. Constant dyspepsia is as liable to cause as to be caused by "a general neurotic disposition."

Our army of neurasthenics is an ever-diminishing one, and as a disinterested observer of a very large number of surgical operations, I shall never make the diagnosis of "gastric neurosis" with complete assurance, nor hear it made without a lurking suspicion that some day surgery will "inconveniently confront us" with something more tangible than our own maxims, and a very solid basis for the hypochondriacal disorder.

In a very great measure the patient is his or her own, and our best guide, if, being of sound mind and appreciating fully the risks, such an one comes deliberately to the conclusion that the pain, vomiting, discomfort and emaciation make life no longer worth living. Then, in the absence of other contra-indications, we are amply justified in advising, if not gastro-enterostomy, at least an exploratory laparotomy.

One last word on the after-treatment. After a gastro-enterostomy has been performed by any of the present methods, there are constantly being reported disappointments of one sort or another, and various mechanical explanations have been given, vicious circles talked of, and numerous twists and kinks given to the gut in order to circumvent the difficulties. In point of fact, is the after-treatment always sufficiently cautiously proceeded with ? My experience with the operation may be exceptional, but I have not encountered as yet one of these results, perhaps because the patients have usually continued for about two months after operation a course of dieting and treatment precisely as though they were recovering from acute ulcer with hæmatemesis.

